# Identifying the missing proteins in human proteome by biological language model

**DOI:** 10.1186/s12918-016-0352-6

**Published:** 2016-12-23

**Authors:** Qiwen Dong, Kai Wang, Xuan Liu

**Affiliations:** 1 Institute for Data Science and Engineering, East China Normal University, Shanghai, 200062 People’s Republic of China; 2 Key Laboratory of Network Oriented Intelligent Computation, Harbin Institute of Technology Shenzhen Graduate School, Shenzhen, 518055 People’s Republic of China; 3 College of Animal Science and technology, Jilin Agricultural University, Changchun, 130118 People’s Republic of China; 4 College of Engineering, Shanghai Ocean University, Shanghai, 201303 People’s Republic of China

**Keywords:** Human proteome, Missing protein, Biological language model

## Abstract

**Background:**

With the rapid development of high-throughput sequencing technology, the proteomics research becomes a trendy field in the post genomics era. It is necessary to identify all the native-encoding protein sequences for further function and pathway analysis. Toward that end, the Human Proteome Organization lunched the Human Protein Project in 2011. However many proteins are hard to be detected by experiment methods, which becomes one of the bottleneck in Human Proteome Project. In consideration of the complicatedness of detecting these missing proteins by using wet-experiment approach, here we use bioinformatics method to pre-filter the missing proteins.

**Results:**

Since there are analogy between the biological sequences and natural language, the *n*-gram models from Natural Language Processing field has been used to filter the missing proteins. The dataset used in this study contains 616 missing proteins from the “uncertain” category of the neXtProt database. There are 102 proteins deduced by the *n*-gram model, which have high probability to be native human proteins. We perform a detail analysis on the predicted structure and function of these missing proteins and also compare the high probability proteins with other mass spectrum datasets. The evaluation shows that the results reported here are in good agreement with those obtained by other well-established databases.

**Conclusion:**

The analysis shows that 102 proteins may be native gene-coding proteins and some of the missing proteins are membrane or natively disordered proteins which are hard to be detected by experiment methods.

## Background

Proteins play important roles in biology. The Human Genome Sequence Project [1] provides a comprehensive compendium about all the human protein encoding genes. However, due to the diversity of proteins and the under-development of current proteomics technology, there are many proteins which have not been identified and annotated.

The Human Proteome Project (HPP) [2] was launched by the Human Proteome Organization (HUPO) in 2011, which contains the Chromosome-centric HPP (C-HPP) [3] and Biology/Disease-Driven HPP (B/DHPP) [4]. This project tries to identify as more proteins as possible with the goal of covering all human protein-encoding genes. This great goal is cooperated by an international associations contains 25 members [5]. The baseline metrics for the HPP contains five annually updated data resources [5]: the Ensembl database [6] provides the possible genes coding proteins; Peptide Atlas [7] and GPMdb [8] separately screen high confident proteins from mass spectrometry data; the Human Protein Atlas [9] is in charge of extracting proteins by antibody-based research; and finally neXtProt [10] collects all human proteins and assigns confidence level (PE 1-5) by protein expression evidence. Proteins at the PE1 level are identified at protein expression level by mass spectrometry, immunohistochemistry, 3D structure, and/or amino acid sequencing. The proteins at PE2 level is detected by transcript expression but not by protein expression. At PE3 level, there are no protein or transcript evidence, but have homologies represented in related species. Proteins at PE4 level are speculated from gene models. Finally, the protein sequences at PE5 level are generated from “dubious” or “uncertain” genes which seemed to have some protein-level evidence in the past but such identifications are doubtful by curation.

Much progress has been achieved since 2011 by the proteomics community and the HPP. Based on the curation of neXtProt [11] database, currently 82% of the protein-coding genes in human have protein expressions with high-confidence. However, there are 3, 564 genes at levels PE2-5 which have no or insufficient evidence of identification by any experimental methods and are thus named as “missing proteins” [11]. Many of these missing proteins are hard to be detected because of low abundance, poor solubility, or indistinguishable peptide sequences within protein families. The missing of such a significant amount of proteins marks a significant problem about our current understanding of the human proteome, with particularly important questions including, e.g., whether these proteins are essential to the cell functions and if yes what biological roles they play in cell and why they are not detectable by the current instruments of both transcription and translation levels. Thus identifying the missing proteins will be a challenging task.

Previous study has shown that there are analogies between biological sequences and natural language. In linguistics, some words and phrases can form a meaningful sentence; in biology, the tactic nucleotides denote gene, and the fixed protein sequences can determine its structure and function. Tsonis [12] discussed that whether DNA is a language or not. Many linguistic approaches have been used in computational biology [13–15]. Ganapathiraju et al. [16] analyzed the language feature of whole-genome protein sequence. Many techniques of Natural Language Pprocess have been used in bioinformatics, such as protein domain recognition based on language modeling [17], dictionary-driven protein annotation [18], protein remote homology detection by latent semantic analysis [19–22], identification of DNA-binding protein [23, 24], and so on.

In this study, the missing proteins in human proteome are identified by using biological language model. The amino acid *n*-gram models for human and non-human protein sequences are constructed. These models are subsequently used to discriminate whether the missing proteins are natively gene-coding proteins in human or not. The identified proteins are then analyzed by their predicted structures and functions, annotation from neXtProt database [10], HGNC database [25] and other mass spectrometry dataset [26].

## Methods

### Datasource

The native gene-coding proteins are downloaded from Swiss-Prot database [27]. To construct the reliable models, only the protein sequences with reviewed items are selected. Totally, there are 14565 human proteins and 70854 non-human proteins. The redundant sequences are then filtered by using CD-HIT program [28] with the sequence identity threshold of 90%. Finally, we get 14189 human proteins and 59060 non-human proteins, which are used to build the *n*-gram model for human and non-human respectively.

The “dubious” or “uncertain” missing proteins with confidence code “PE5” are extracted from the neXtProt database [10] that was released at Sep. 19, 2014. There are in total 616 proteins in this category with length ranging from 21 to 2, 252 residues. The structures of these proteins are predicted by using I-TASSER software [29]. The functions including the EC number, the GO terms and the binding sites are predicted by using COFACTOR software [30]. Both I-TASSER and COFACTOR are run in non-homology mode where all the homologous structures identified with sequence identities greater than or equal to 30% are removed. The subcellular localization is predicted by using Hum-mPLoc [31].

### Biological language models to discriminate the native gene-coding human and non-human proteins

The protein sequences are composed of 20 native amino acids, while in natural language, the sentences are comprised by words. Such similarity has draw researchers attention and the language features of DNA and protein sequence have been investigated extensively [32–34]. In this study, the methods from natural language processing, especially, the statistical natural language processing methods which have been successfully solve many of the natural language task [35], are applied to identify the missing proteins. Formally, the problem of identifying missing proteins can be described as following: given a protein *P* = (*a*_1_, *a*_2_, …, *a*_L_), is this protein a human protein or not:

1 $$ P\left(H\Big|P\right)>P\left(NH\Big|P\right) $$

where *P*(*H|P*) and *P*(*NH*|*P*) represent the probability of this protein belongs to human and non-human. The above equation can be converted by conditional probability formulae:

2 $$ \frac{P(H)P\left(P\Big|H\right)}{P(P)}>\frac{P(NH)P\left(P\Big|NH\right)}{P(P)} $$

Since the denominator is the same, it can be ignored during the comparison. *P*(*H*) and *P*(*NH*) are the prior probability of human and non-human proteins, and can be estimated from the training dataset by using maximum likelihood estimation based on the number of human and non-human protein. By applying the conditional probability formulae repeatedly, the probability of *P*(*H*|*P*) or *P*(*NH*|*P*) can be decomposed into:

3 $$ \begin{array}{l}P\left(P\Big|H\right)=P\left({a}_1\dots {a}_L\Big|H\right)\hfill \\ {}=P\left({a}_1\Big|H\right)P\left({a}_2\dots {a}_L\Big|H,{a}_1\right)\hfill \\ {}=P\left({a}_1\Big|H\right)P\left({a}_2\Big|H,{a}_1\right)P\left({a}_3\dots {a}_L\Big|H,{a}_1,{a}_2\right)\hfill \\ {}=P\left({a}_1\Big|H\right){\displaystyle {\sum}_{i=2}^L}P\left({a}_i\Big|{a}_1\dots {a}_{i-1},H\right)\hfill \end{array} $$

The *n*-gram model supposes that the occurrence of each word is only dependent on the previous *n*-1 words, so the above equation can be recalculated as:

4 $$ P\left(P\Big|H\right)\approx {\displaystyle {\sum}_{i=n}^L}P\left({a}_i\Big|{a}_{i-n+1}\dots {a}_{i-1},H\right) $$

where the conditional probability can be estimated by maximum likelihood estimation:

5 $$ P\left({a}_i\Big|{a}_{i-n+1}\dots {a}_{i-1}\right)=\frac{C\left({a}_{i-n+1}\dots {a}_i\right)}{C\left({a}_{i-n+1}\dots {a}_{i-1}\right)} $$

where *C*(*a*_*i*_ *…a*_*j*_) is the number of occurrence of amino acid sequence *a*_*i*_ *..a*_*j*_.

The same procedure can be applied to construct the *n*-gram model of non-human proteins. The missing proteins can be identified by using *n*-gram models to get whether it’s native human proteins or not.

## Results and discussions

Based on the *n*-gram models, there are 102 proteins in the neXtProt “PE5” category which have high probabilities to be native human proteins. In the following sections, these proteins are analyzed by the predicted structure and function and annotations from other databases.

### The structure and function analysis of the high-probability proteins

**Fig. 1:**
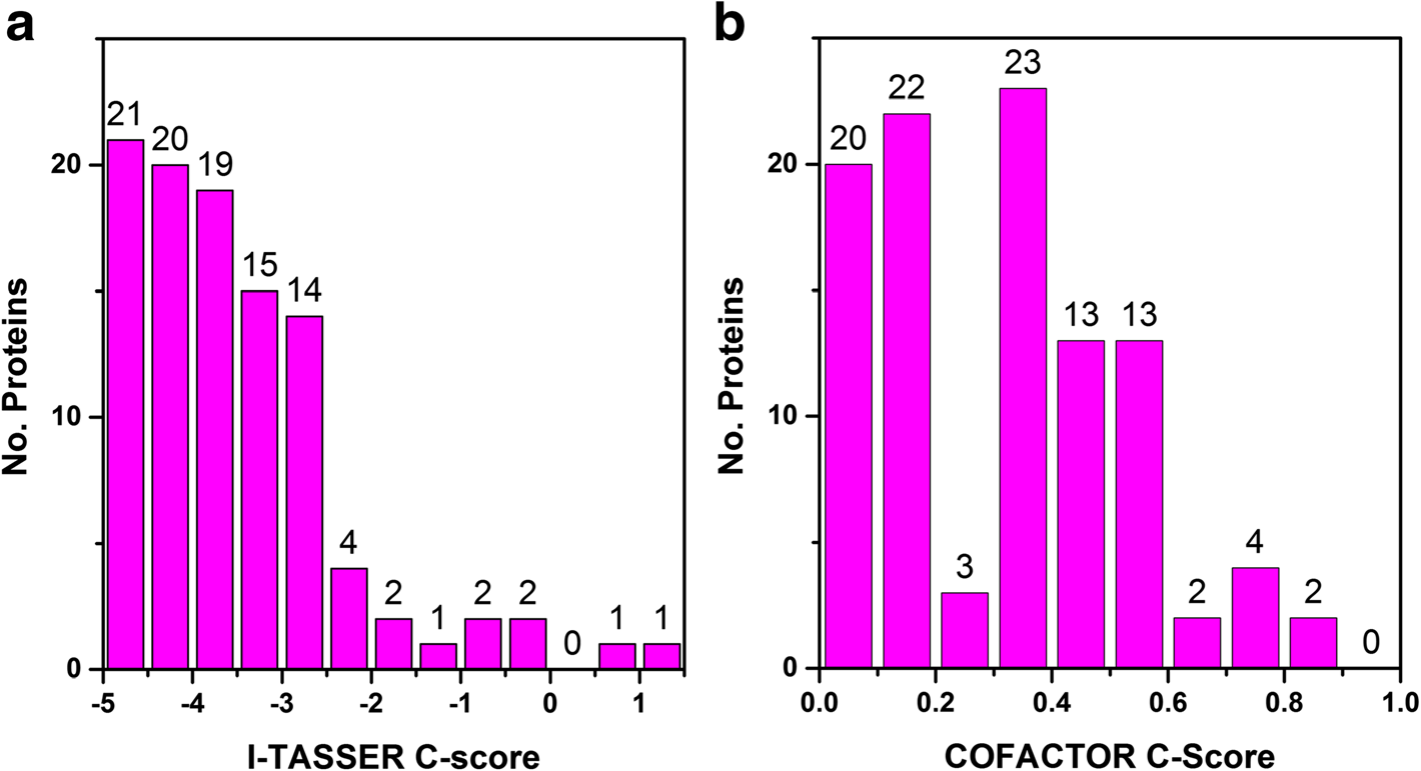
The distribution of I-TASSER (**a**) and COFACTOR (**b**) C-score of the identified missing proteins

Since the missing proteins have not been identified by experiment methods, their structures and functions are currently unknown. In this study, the structures and functions of the high-probability proteins inferred by *n*-gram models are predicted by I-TASSER and COFACTOR software and the confidence scores outputted by the software are used to indicate the reliability of the prediction. The I-TASSER confidence score (C-score) is computed by the accuracy of the threading programs and the simulation results of the structural assembly process. The value range is between -5 and 2, where the higher the C-score value is, the more confident the corresponding model is, and vice-versa. A model with I-TASSER C-score larger than -1.5 means that the structure topology is correct. The confidence score (C-score) of COFACTOR is calculated based on the confidence score of the structure prediction and the similarity between the predicted models and their native structures in the PDB. The COFACTOR C-score are normalized between 0 and 1, where a large value indicates a good prediction. Figure 1 shows the distribution of I-TASSER and COFACTOR C-score of the 102 high -probability missing proteins. The number of foldable proteins (with C-score higher than -1.5) are less than that of the un-foldable proteins (with C-score lower than -1.5) which is also confirmed in our previous study about missing proteins [36]. The reason for this phenomenon may be that the missing proteins are not gene-coding proteins or there is no homology templates used during prediction. Based on the results of structure prediction, there are 7 foldable proteins whose I-TASSER C-score are larger than the foldable threshold (-1.5). These proteins have good structure models with no homologous templates used during prediction, which means that they may be gene-coding proteins. Most of the COFACTOR C-scores are distributed between 0.3 and 0.6, where 8 proteins have very high COFACTOR C-score. As shown in the figure, the COFACTOR C-score for most of the missing proteins are larger than 0.2, which indicates a good function prediction based on experience evaluation.

### Structural topology analyses of the I-TASSER models

**Fig. 2:**
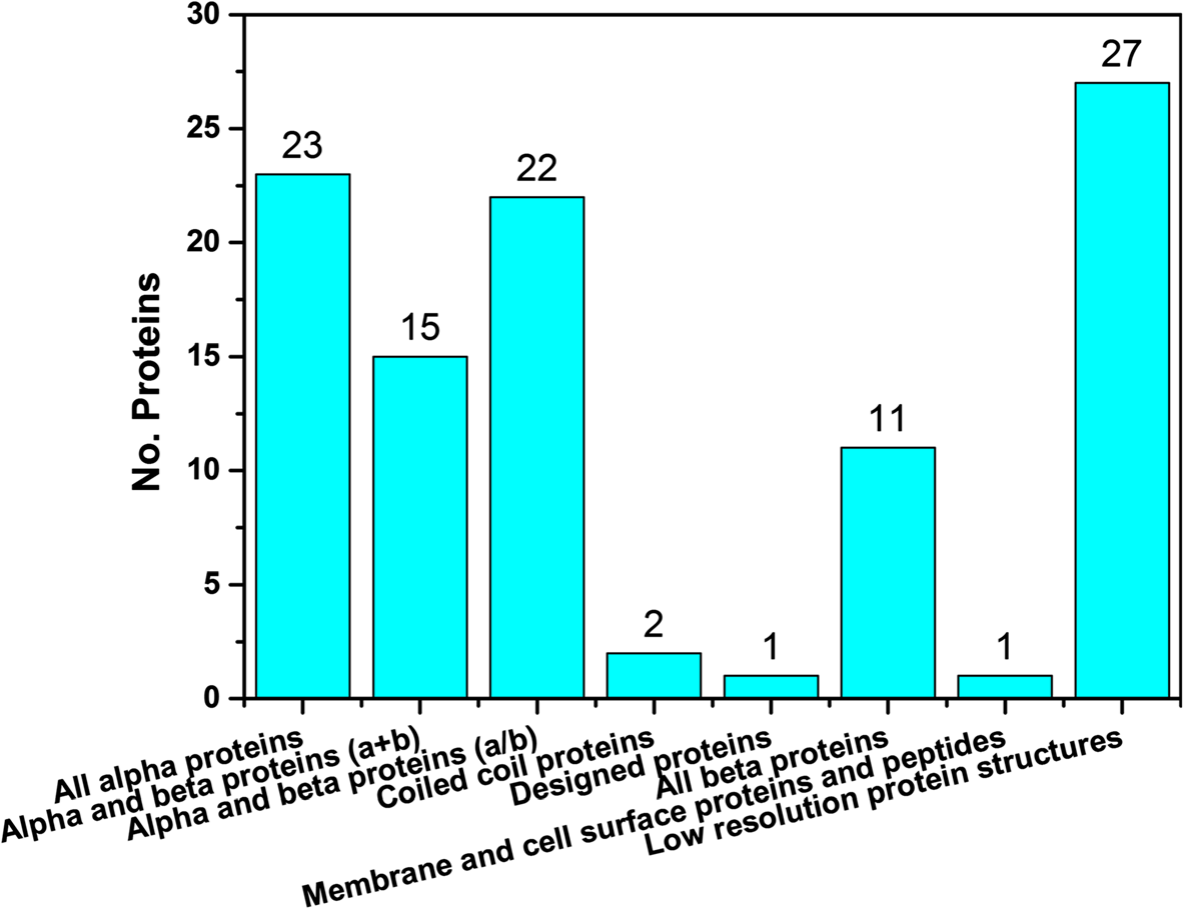
Relative frequency distribution of SCOP classes for the identified missing proteins

The SCOPe library [37] is used as the classification criterion of structure topology, which is an extended structure library integrated from the standard SCOP [38] and ASTRAL [39] databases. The structure class of the I-TASSER model is assigned as the corresponding structure class of the SCOPe domain which has the highest structural similarity with the model. The structure alignment program TM-align [40] is used to calculate the TM-score between the I-TASSER model and all structural domains from SCOPe. If there are multiple domains in target model, we selected the domain that has the maximum TM-score to SCOPe domain. Figure 2 shows the distribution of the SCOPe class of the identified missing proteins. It is interesting that some missing proteins have structure topology in ‘membrane and cell surface proteins and peptides’ and ‘coiled coil proteins’ class. Such phenomenon is reasonable since these kind of proteins are difficult to be identified by experiment method.

### Evaluation of the function base on gene ontology

**Fig. 3:**
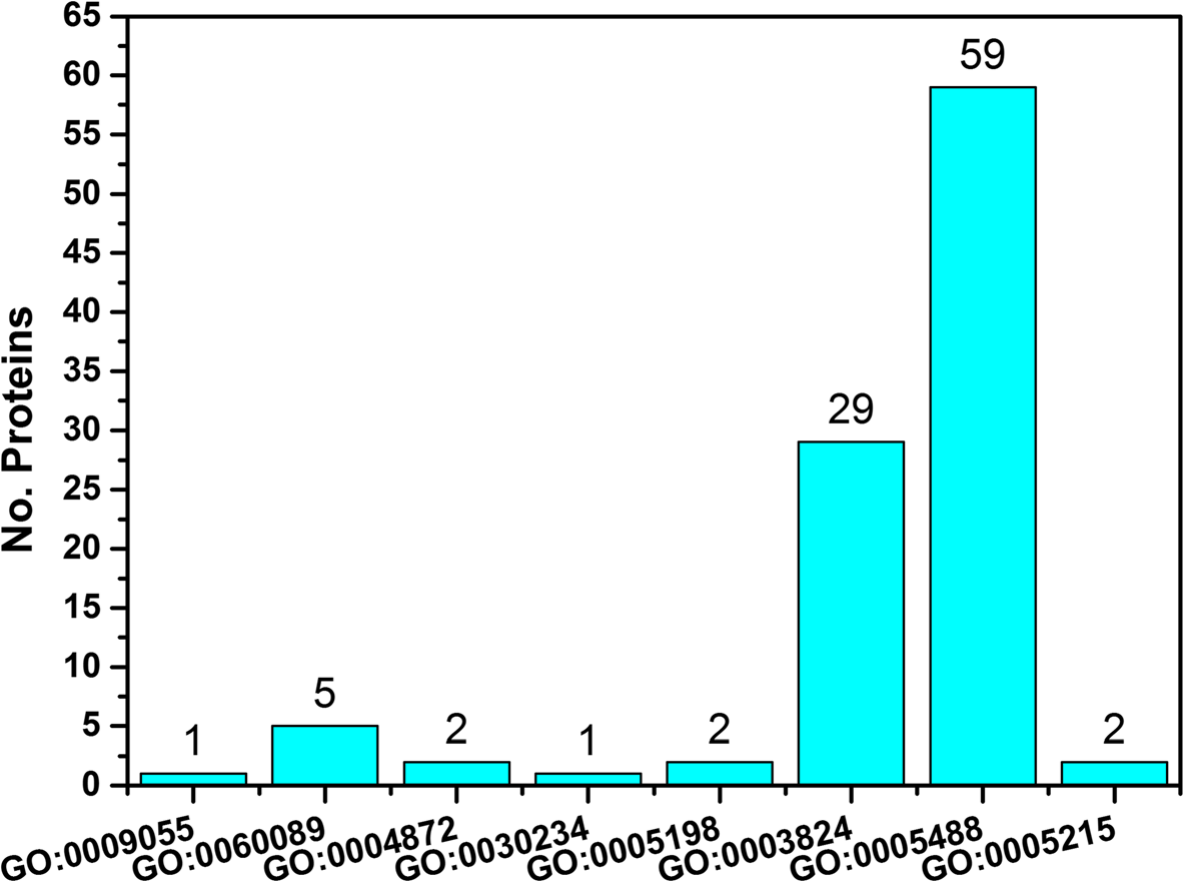
The distribution of predicted GO items from the fist level of molecular function

The GO molecular function of the high-probability missing proteins is predicted by the COFACTOR package and the number in each GO item is shown in Fig. 3. As shown in the Fig. 3 GO terms come from the first level of GO molecular function. Most of the high-probability missing proteins have the GO function of ‘binding’ (GO:0005488) and ‘catalytic activity’ (GO:0003824). However some missing proteins may be membrane proteins since they have the GO function of ‘transporter activity’ (GO:0005215) and ‘receptor activity’ (GO:0004872). The results are consistent with the structural topology analysis in which there are many membrane proteins in the missing proteins.

### Comparision of subcellular localization

**Fig. 4:**
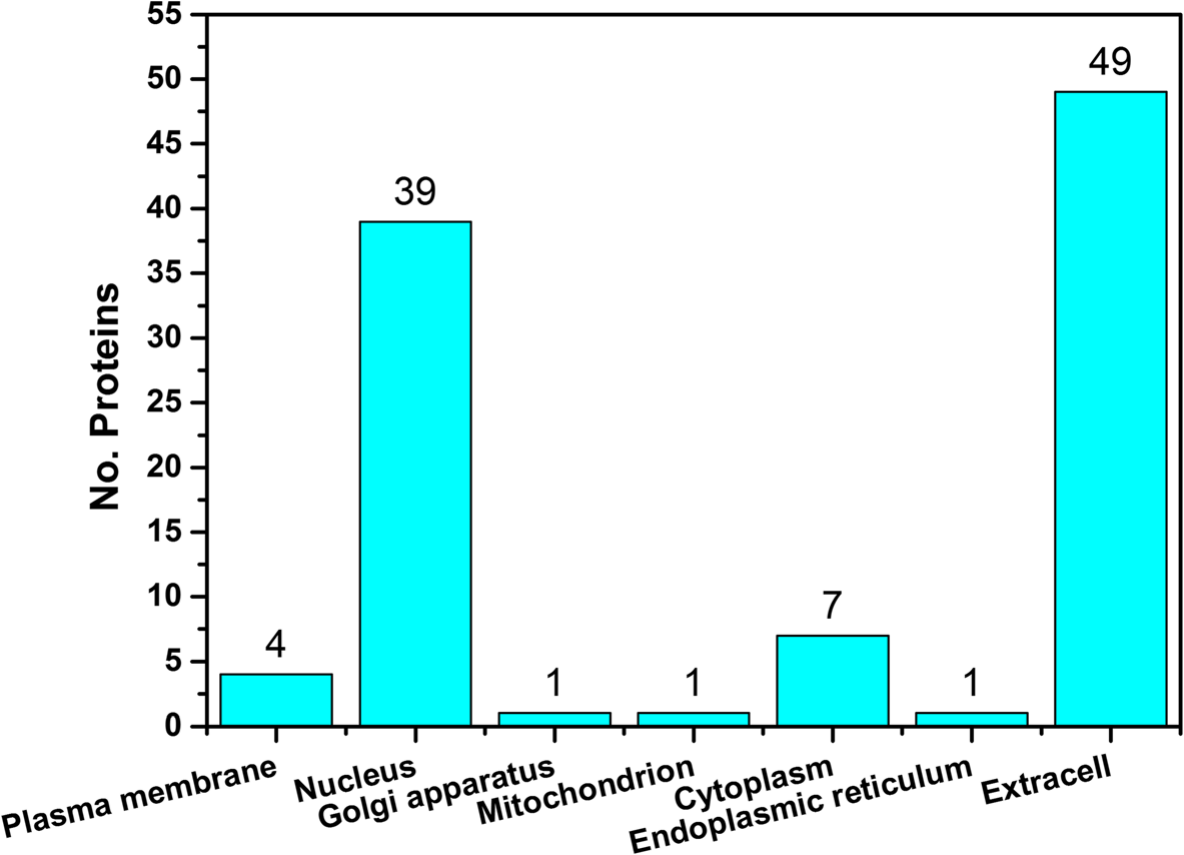
The distribution of subcellular localizations for the identified missing proteins

The subcellular localizations of proteins are critical for their biological functions. The Hum-mPLoc 2.0 program [31] is used to predict the subcellular localizations of the high-probability missing proteins. The types of subcellular localizations and the number of proteins in each types is illustrated in Fig. 4. Most of the proteins are located at extracellular and nucleus. The missing proteins are also observed at plasma membrane, which is also confirmed by the results from the structural topology analysis and function predictions.

### HGNC mapping analysis

The HGNC [41] database is in charge of assigning a unique symbol and name for each gene loci from human genome. Most of the HGNC data are manually collected and carefully checked [41]. The information from HGNC gene loci provides valuable resource to identify the missing proteins. Based on the Gene mapping, 71 out of the 102 high-probability missing proteins can be mapped to one or more HGNC items. We collected the corresponding gene loci types for the 71 missing proteins and counted the number of missing proteins in each loci type. The results are shown in Table 1. There are 9 proteins confirmed by HGNC with gene loci type “gene with protein product”. There are 26 pseudogenes. Since pseudogenes are the products of evolution. They usually have homologous proteins. That’s the reason why there are many pseudogenes in the missing proteins.

**Table 1:** The gene loci types and the number of proteins for the high-probability missing proteins after hgnc mapping

Gene loci type	No. missing proteins
Gene with protein product	9
Pseudogene	26
RNA, long non-coding	30
Unknown	6

### Consistence analysis with other mass spectrometry dataset

Mass spectrometry is currently one of the efficient method to identify protein peptides. There are many mass spectrometry data deposited in public database, such as PeptideAtlas [42] and GPMDB [8]. The sketch of human proteome is drawing by mass spectrometry data [26, 43]. Recently Kim et al [26] reported that about two-third (2535/3844) of the “missing proteins” [11] have been identified. Actually the “missing proteins” used by Kim et al. are constituted by the neXtProt proteins with evidence codes of “PE2”, “PE3” or “PE4”. This paper aims to identify the “PE5” missing proteins. By RefSeq [44] mapping, we found that there are 41 “PE5” proteins which are also in Kim’s dataset. Among these 41 missing proteins, there are 6 proteins are foldable based on the structure prediction results in non-homology mode. These results indicate that our finding are in good consistent with Kim’s results.

## Conclusion

In this study, the human gene-coding proteins currently undetected are identified by using biological language models. The amino acid *n*-gram models of human and non-human proteins are constructed. These models are then used to identify the “uncertain” missing proteins with evidence code “PE5” from neXtProt database. The results show that 102 high probability proteins may be gene-coding proteins. The structure, function and subcellular localization of these proteins are then inferred by using the advanced programs. The identified missing proteins are then analyzed with the annotation from other database. Without using homology templates, 7 proteins have correct structure topology with I-TASSER C-score larger than -1.5. The predicted functions are mainly within GO items ‘binding’ (GO:0005488) and ‘catalytic activity’ (GO:0003824). 9 missing proteins are confirmed by HGNC with gene loci type “gene with protein product”. 6 missing proteins are also detected by mass spectrometry experiment. The analysis also shows that many of the unknown proteins are membrane or natively disordered proteins which are difficult to be detected. The identified missing proteins need to be further validated by experimental approach. The results in this study provides valuable complementary resource for the human proteome.
